# Outcomes and Safety of Retrograde Intra-Renal Surgery for Renal Stones Less Than 2 cm in Size

**DOI:** 10.5812/numonthly.2211

**Published:** 2012-03-01

**Authors:** Christopher C. K. HO, Tan Guan Hee, Goh Eng Hong, Praveen Singam, Badrulhisham Bahadzor, Zulkifli Md Zainuddin

**Affiliations:** 1 Urology Unit, Department of Surgery, University Kebangsaan Malaysia Medical Centre, Kuala lumpur, Malaysia

**Keywords:** Ureteroscopy, Calculi, Kidney

## Abstract

**Background:**

Retrograde intra-renal surgery (RIRS) has been used to remove stones of less than 2 cm in the kidney. However, its role is not well defined.

**Objectives:**

The objective of this study was to evaluate the outcomes and safety of RIRS, used either as a primary or secondary procedure, and to analyze factors predicting the stonefree rate (SFR).

**Patients and Methods:**

A retrospective analysis was performed on data from patients who underwent RIRS over a 10-year period (2002–2012). Stone size was measured as the surface area and was calculated according to the EAU guidelines. In cases of multiple stones, the total stone burden was calculated as the sum of each stone size. Stone burden was then classified as ≤ 80 mm^2^ or > 80 mm^2^. RIRS was classified as primary procedure or secondary procedure (after failed extracorporeal shockwave lithotripsy or percutaneous nephrolithotripsy).Stone clearance was defined as a complete absence of stones or stones < 4 mm, which were deemed insignificant on ultrasonography and plain radiography.

**Results:**

The overall SFR for renal stones treated with RIRS in our center was 55.4%, and the complication rate was 1.5%, which consisted of one case of sepsis. The only factor affecting SFR in this study was the indication for RIRS. When performed as a primary operation, RIRS showed a significantly better SFR (64.3%). The SFR for lower pole stones was only 44.4%. There were no statistically significant effects of stone burden, radio-opacity, or combination with ureteral stones on SFR.

**Conclusions:**

RIRS should be used as the primary treatment for renal stones whenever possible.

## 1. Background

The use of flexible ureteroscopy for the management of intra-renal stones (retrograde intra-renal surgery) was introduced 20 years ago and offered urologists an alternative to existing modalities, such as extra-corporeal shockwave lithotripsy (ESWL) and percutaneous nephrolithotripsy (PCNL). Lower pole renal stones are the most challenging and have poor stone-free rates (SFR) (1). They often require multiple procedures to achieve complete stone clearance. The European Association of Urology (EAU) guidelines recommend the use of percutaneous nephrolithotripsy (PCNL) for lower pole stones >2 cm and less invasive modalities, such as ESWL or retrograde intra-renal surgery (RIRS), for stones < 2 cm (1). However, it remains unclear which is the superior modality. To investigate this, several studies have directly compared the different modalities using a primary outcome of SFR.

The use of ESWL as primary therapy for lower pole stones has an SFR of approximately 37%–59% (2). In contrast, the SFR of lower pole stones treated primarily with RIRS is 75%–80% (1, 3, 4). However, in a prospective study evaluating ESWL versus RIRS in the management of lower-pole stones < 1 cm in size, the SFRs were not significantly different (35% and 50%, respectively).(5). For stones 1.5–2 cm in size, Omer et al. found that RIRS had an SFR of 89.2% (6). Some studies have even used RIRS for treating stones >2 cm. The results showed that at least 3 RIRS procedures are needed to achieve 93% SFR (7). The use of RIRS as a secondary procedure after failed ESWL is not appealing. Previous reports comparing primary RIRS to secondary RIRS after a failed ESWL revealed a lower SFR among the latter group (4, 8).

In view of the mixed results, guidelines have failed to recognize RIRS as a primary modality for the treatment of renal stones measuring 1–2 cm. However, there are circumstances that make RIRS a favorable primary procedure due to its higher SFR in a single session. Such circumstances include patients with bleeding diathesis, gross obesity, concomitant ureteric stones, musculo-skeletal deformity, and occupations that require complete stone clearance (i.e., pilots) (9).

## 2. Objectives

Here, we report our experience using RIRS for treating renal stones. We evaluated the outcomes and safety of RIRS based on its use either as a primary or secondary procedure and analyzed factors predicting SFR.

## 3. Patients and Methods

We performed a retrospective study of all patients who underwent RIRS over a 10-year period (2002–2012) at University Kebangsaan Malaysia Medical Center (UKMMC). Patients whose records were incomplete or who failed to follow-up were excluded. All patients had a pre-operative intravenous urography (IVU) or computed tomography urography (CTU) prior to the procedure. No patients failed to return for a follow-up visit. Stone size was assessed as the surface area and was calculated according to the EAU guidelines (1). In cases of multiple stones, the total stone burden was taken as the sum of each stone size. Stone burden was then classified as 80 mm^2^ or less and greater than 80 mm^2^. RIRS was classified as the primary procedure or as the secondary procedure (after a failed ESWL). The case notes and operative records of the patients were reviewed.

Preoperative antibiotics were administered upon the induction of general anesthesia. RIRS was performed using a flexible ureterorenoscope size 3.7F with a 270º-angle deflection (Karl Storz, Endoscopes, Culver City, CA, USA). A holmium 20W Versa Pulse Power Suite laser lithotripter (Lumenis Ltd, Santa Clara, CA, USA) was used for the fragmentation of stones. An access sheath was used in all cases. Double J stents (Microinvasive, 6F, 24 cm, Boston Scientific, Watertown, MA, USA) were inserted in all cases of RIRS and were removed postoperatively within 4 weeks. The stones were fragmented with a holmium: yttrium-aluminum-garnet (Ho: YAG) laser until they were deemed small enough to pass spontaneously. Stone extraction using a basket was performed for larger fragments only.

Patients with radiolucent stones were administered potassium citrate. All patients were encouraged to increase their fluid intake in the absence of contraindications, such as heart failure and renal failure. Postoperative stone evaluations were conducted using plain radiographs and ultrasonography at 1 month and 6 months after treatment. These imaging results were reported by the same radiologist who first diagnosed the stone. Stone clearance was defined as complete stone absence or stones <4 mm, which were deemed insignificant, as seen on ultrasonography and plain radiography.

Chi-square and t-tests were used for statistical analysis where relevant. A P-value of <0.05 was considered statistically significant. Data were analyzed with standard statistical software, SPSS ver. 16.0 (SPSS Inc., Chicago, IL, USA).

## 4. Results

Of the 66 patients with renal stones who underwent RIRS, 60 (90.9%) had complete data. Of these, 5 had bilateral renal stones. Therefore, there were a total of 65 procedures. Thirty-two patients were males, and 28 were females. The median age was 49.4 years (range, 15 to 72 years). For ethnic distribution, there were 36 Malays, 16 Chinese, and 8 Indians. Thirty-five of the 65 cases of RIRS were performed on the left kidney, and 30 cases were performed on the right kidney. For stone location, there were 27 stones in the lower pole (regardless of co-existing stones in other locations) and 38 stones in other locations (Table 1).

**Table 1:** Patient Demographics and Baseline Characteristics of Renal Stones

Variables	
Number of patients	60
Number of RIRS	65
Median age, range, y	49.4 (15–72)
Gender	
Male	32
Female	28
Laterality	
Left	35
Right	30
Location, No. (%)	
Lower pole with or without others	38 (58.5)
Others	27 (41.5)
Cumulative stone burden, No.(%)	
Less than 80 mm ^2^	44 (67.7)
80 and above mm ^2^	21 (32.3)
Radio-opacity, No. (%)	
Yes	51 (78.5)
No	14 (21.5)
Combination with ureteral stone, No. (%)	
Yes	10 (15.4)
No	55 (84.6)
Indication, No. (%)	
Primary RIRS	42 (64.6)
Secondary RIRS (failed ESWL)	20 (30.8)
Secondary RIRS (failed PCNL)	3 (4.6)

At least two-thirds of the patients undergoing RIRS had stone burdens less than 80 mm^2^, and the other one-third had burdens of 80 mm^2^ and above. Almost 80% of all stones were radio-opaque. RIRS combined with stone fragmentation in the ureter constituted 15.4% of the cases. Approximately two-thirds of the RIRS procedures were primary cases (64.6%), while secondary cases comprised 35.4%. The majority of the secondary RIRS procedures were performed after failed ESWL (30.8%), and the remaining 4.6% were after failed PCNL. Among the analyzed variables, only one factor was significantly related to stone clearance. Primary RIRS was almost twice as likely to result in total stone clearance compared to secondary RIRS (clearance rates, 64.3% and 39.1%, respectively, P = 0.045) (Table 2). The SFR for lower-pole stones was only 44.4%. There were no statistically significant differences in SFR in terms of stone burden, radio-opacity, and combination with ureteral stone. There were no major perioperative complications associated with this procedure. Only one case of sepsis was recorded (1.5%). This was a mild case of febrile urinary tract infection that resolved after a short course of antibiotics. No other complications, such as ureteric injury or perforation, were reported in the current study.

**Table 2:** Analysis of Variables and Immediate Post-Operative Stone-Free Rates

Variables	Number of Cases	Stone-Free Rate, No. (%)	P value
Indication			0.045
Primary RIRS	42	27 (64.3)	
Secondary RIRS	23	9 (39.1)	
Location			0.107
Lower pole with or without others	27	12 (44.4)	
Others	38	24 (63.2)	
Cumulative stone burden (mm^2^)			0.273
Less than 80	44	26 (59.1)	
80 and above	21	10 (47.6)	
Radio-opacity			0.141
Yes	51	26 (51.0)	
No	14	10 (71.4)	
Combination with ureteral stone			0.236
Yes	10	4 (40.0)	
No	55	32 (58.2)	

## 5. Discussion

In this study, the overall SFR was 55.4% following RIRS for renal stones less than 2 cm in size. This figure is low compared to previous studies, which reported SFRs of 69.7%–89.2% (2, 6). Several factors have been shown to influence SFR. In another analysis of 66 cases of RIRS, lower pole stones, greater cumulative stone burden, and more total stones all reduced SFR in RIRS (2).

In the present study, stone position did not affect the SFR. This is consistent with another study by Perlmutter et al., who found no significant differences in the SFR between stones in different positions (10). When lower-pole stones were analyzed separately, the SFR was 44.4%. This outcome is comparable to another study by Pearle et al. that reported an SFR of 50% for lower-pole stones measuring 1 cm or less (5). A greater cumulative stone burden also failed to affect the SFR in the current study. Lim et al. found that a cumulative stone burden of >150 mm^2^ was associated with a significantly lower SFR (2). We chose a cut-off value of 80 mm^2^, because we only included stones that were < 2 cm. A stone burden of 80 mm^2^ corresponds to a stone dimension of approximately 1 cm in diameter, which is half of the largest stone size included in this study (1). Neither radio-opacity of the stones nor combined renal and ureteral stones affected the SFR in our study. This result is similar to that of Lim et al., who also found that these parameters did not affect SFR.

In the present study, the only factor affecting SFR was whether the RIRS was performed as a primary or secondary procedure. Cases where RIRS was used as the first treatment modality were considered primary. Cases where RIRS was used in patients previously treated with failed ESWL were considered secondary. When performed as a primary procedure, RIRS resulted in a better SFR. However, the reason for this observation is unclear. It is possible that stones that could not originally be cleared with ESWL introduced a selection bias in this analysis. It has been shown that ESWL and RIRS have the same efficacy in treating lower pole stones of 1 cm or less in size (5). Lim et al. initially found the SFR to be better in primary RIRS, but this results was not significant in a subsequent multivariate analysis (2). One explanation for the lower SFR in the present study is that the stones we encountered may have been harder. Ideally, we would like to measure the composition of the stones treated in this center; however, this service is not readily available. With widespread use of non-contrasted spiral computed tomography (CT), some studies have correlated the stone features on CT with their composition (11). Due to the inherent limitations of a retrospective study, we were unable to uniformly obtain CT features of the stones treated in this study. However, it would be useful to study the association between stone composition and SFR in RIRS.

The current study found a complication rate of 1.5%. In another similar study, there was a 6% complication rate related to RIRS. Among the complications reported were minor ureteric injury, febrile urinary tract infection, and paralytic ileus. It was concluded that RIRS is a safe and effective modality for treating renal stones. The overall SFR for renal stones treated with RIRS in our center was 55.4%. The only factor that significantly affected SFR in this study was the indication for RIRS. When the procedure was performed as a primary operation, it showed a significantly better SFR (64.3%). Therefore, RIRS should be used as a primary mode of treatment for renal stones whenever possible.
